# Targeting Tumor-Associated Macrophages as a Potential Strategy to Enhance the Response to Immune Checkpoint Inhibitors

**DOI:** 10.3389/fcell.2018.00038

**Published:** 2018-04-04

**Authors:** Luca Cassetta, Takanori Kitamura

**Affiliations:** ^1^Medical Research Council Centre for Reproductive Health, Queen's Medical Research Institute, Edinburgh, United Kingdom; ^2^Royal (Dick) School of Veterinary Studies and the Roslin Institute, University of Edinburgh, Edinburgh, United Kingdom

**Keywords:** tumor microenvironment, immunotherapy, checkpoint inhibitor, CD8^+^ T cell, macrophage, TAM, tumor immunology

## Abstract

Inhibition of immune checkpoint pathways in CD8^+^ T cell is a promising therapeutic strategy for the treatment of solid tumors that has shown significant anti-tumor effects and is now approved by the FDA to treat patients with melanoma and lung cancer. However the response to this therapy is limited to a certain fraction of patients and tumor types, for reasons still unknown. To ensure success of this treatment, CD8^+^ T cells, the main target of the checkpoint inhibitors, should exert full cytotoxicity against tumor cells. However recent studies show that tumor-associated macrophages (TAM) can impede this process by different mechanisms. In this mini-review we will summarize recent studies showing the effect of TAM targeting on immune checkpoint inhibitors efficacy. We will also discuss on the limitations of the current strategies as well on the future scientific challenges for the progress of the tumor immunology field.

## Introduction

Solid tumors are “aberrantly developing organs” in the body initiated by oncogenic mutations, which causes infiltration of different population of immune cells. A recent study shows that high number of cytotoxic lymphocytes such as natural killer (NK) or CD8^+^ T cells in the tumors correlate with favorable prognosis, whereas high infiltration of myeloid cells such as eosinophils, tumor-associated macrophages (TAM) and neutrophils is associated with poor prognosis in most solid tumors (Gentles et al., 2015). Since TAM is one of the most abundant cell types in tumor (Qian and Pollard, 2010), several metanalyses further evaluated the correlation of TAM infiltration with clinical stage, overall survival and recurrence free survival in different cancers, and indicated that high infiltration of TAM correlates with poor overall survival in breast, gastric, oral, ovarian, bladder and thyroid cancers, but not in colorectal cancer (Zhang et al., 2012; Guo et al., 2016; Mei et al., 2016; Yin et al., 2017; Zhao et al., 2017). These studies indicate that the poor prognostic outcome of a neoplastic lesion is determined not only by the type of mutation occurred but also by the tumor stromal composition especially immune cells, i.e., the recruitment and activation of cytotoxic lymphocytes (e.g., CD8^+^ T cells) can suppress lethal tumor development whereas the infiltration of TAMs promotes it. Better understanding of these tumor suppressing and tumor promoting cells is thus essential to establish efficient cancer immunotherapies. Decades of dogged studies about the CD8^+^ T cell functions have established several immunotherapeutic strategies including cancer vaccination, transfer of *ex vivo* activated CD8^+^ T cells, and administration of cytokines that activate CD8^+^ T cells. Of special note is the success of checkpoint inhibitors that reboot CD8^+^ T cells in the tumors (Farkona et al., 2016; Khalil et al., 2016). In contrast, studies about the effects of immune suppressor cells on these therapies are just started. In this review, we highlight the recent findings about suppressive effects of TAM on checkpoint immunotherapy, and discuss therapeutic potential of a novel immunotherapy combined checkpoint inhibition with TAM intervention.

## The role of macrophages in the immune response in solid tumors

During initiation and progression of solid tumors, mutant and thus potentially immunogenic cancer cells are exposed to and interact with a complex immune system, which will determine the fate of cancer cells.

Cytotoxic lymphocytes such as CD8^+^ T cells and NK cells have potential to detect and eliminate cancer cells by inducing apoptosis. Macrophages are also potentially able to mount a robust anti-tumoral response as they can directly kill cancer cells if properly activated and support the adaptive immune response by presenting tumor antigens and by producing chemokines and cytokines that recruit and activate cytotoxic CD8^+^ T cells and NK cells (Gifford et al., 1986; Brigati et al., 2002). So, if these immune reactions are dominant in the tumor microenvironment, the development of malignant tumors will be suppressed.

However, in many cases the tumor microenvironment alters macrophage functions from the pro-inflammatory (i.e., tumoricidal) to the trophic ones that resemble those of macrophages in the developing tissues (Pollard, 2009; Noy and Pollard, 2014). As a result, these tumor-educated macrophages promote malignant tumor development instead of suppressing it. For example, studies using different mouse models of solid tumors demonstrated that TAM promotes angiogenesis, cancer cell invasion and intravasation in the primary site, as well as extravasation and persistent growth in the secondary site (Qian and Pollard, 2010; Kitamura et al., 2015). Furthermore, several studies suggest that TAM is likely to protect cancer cells from the anti-tumor immune responses. For example, TAM expresses programmed cell death ligand 1 (PD-L1), PD-L2, CD80, and CD86 that restrict CD8^+^ T cell activities upon binding to the immune-checkpoint receptors, programmed cell death protein 1 (PD1) and cytotoxic T-lymphocyte-associated protein 4 (CTLA4) (Noy and Pollard, 2014; Mantovani et al., 2017). It is also reported that macrophages isolated from the mouse and human tumors can directly suppress T cell responses *in vitro* (Ruffell and Coussens, 2015), and that depletion of TAM enhances CD8^+^ T cell-mediated anti-tumor immunity in the mammary tumors in mice under treatment with chemotherapy (DeNardo et al., 2011). Therefore, TAM represents immune suppressor cells in the solid tumors that restrict anti-tumor immune reaction induced by CD8^+^ T cells.

## Checkpoint inhibitors as a novel antitumor therapeutic strategy

One of the most successful approaches to mount CD8^+^ T cell-mediated anti-tumor immune reaction is the administration of checkpoint inhibitors, i.e., blocking antibodies against inhibitory checkpoint receptors (e.g., PD-1 and CTLA4) or ligands (e.g., PD-L1) (Farkona et al., 2016; Khalil et al., 2016). Strikingly there have been successful clinical trials with the immune checkpoint inhibitors that have revealed a great potential of immunotherapies for the treatment of malignant tumors such as melanoma and lung cancers (Sharma and Allison, 2015). However, the majority of patients in most cancers do not fully respond to this type of immunotherapy for reasons still unknown. Although this lack of response could be due to the expression of checkpoint ligands in cancer cells and microbiota composition (Sharma et al., 2017; Gopalakrishnan et al., 2018; Routy et al., 2018), recent studies indicate that expression of PD-L1 in leukocytes rather than tumor cells is essential for PD-L1 blockade-mediated tumor regression (Lin et al., 2018; Tang et al., 2018), which emphasizes the contribution of tumor-infiltrating leukocytes to the insufficiency of checkpoint therapies. It has been reported that certain types of leukocytes such as regulatory T (T_reg_) cell, myeloid-derived suppressor cell (MDSC), and TAM suppress T cell activities and promote tumor progression (Kitamura et al., 2015). Given their abundance in the tumor microenvironment, TAM is suggested as one of the important therapeutic targets to enhance the efficacy of immunotherapies utilizing checkpoint antagonists (Mantovani et al., 2017).

## Targeting TAM potentiates the efficacy of checkpoint inhibitors

One of the efficient strategies to target TAM is the blockade of colony-stimulating factor 1 receptor (CSF1R) that is essential for the recruitment, differentiation, and survival of TAM (Mantovani et al., 2017). In mouse models of solid tumors including colon cancer, breast cancer, and glioblastoma, monoclonal antibodies or small molecule inhibitors against CSF1R reduces the number of TAM and/or changes the phenotype of TAM, which impairs tumor development and progression (DeNardo et al., 2011; Pyonteck et al., 2013; Ries et al., 2014). For example, a CSF1R antagonist PLX397 inhibits the infiltration of TAM into the pancreatic tumor and alters phenotype of the remaining TAM, which results in the modest suppression of the tumor growth in mice that have received orthotopic injection of syngeneic pancreatic cancer cells (Zhu et al., 2014). In this model, a combined treatment of the tumor-bearing mice with anti-PD1 and anti-CTLA4 antibodies also limits the tumor outgrowth by ~50% compared with a vehicle treatment. Importantly, the anti-PD1/anti-CTLA4 treatment in combination with PLX3397 completely blocks the tumor expansion and even regresses the established tumors by 15% (Zhu et al., 2014). These results provide a proof of concept that the TAM targeting improves efficacy of CD8^+^ T cell-based immunotherapies using checkpoint antagonists.

In the pancreatic cancer model, blockade of CSF1R signaling significantly reduces the number of TAM in the tumor as well as mRNA expression of immunosuppressive molecules such as PD-L2, transforming growth factor-β (TGF-β), and arginase-1 (ARG1) in the remaining TAM (Zhu et al., 2014), suggesting that CSF1R inhibition improves checkpoint therapies not only by depletion of TAM but also by reducing their expression of suppressive molecules. It has been reported that TAM isolated from the subcutaneous tumor established by C3 fibrosarcoma express higher level of ARG1 compared with normal splenic macrophages, and suppress T cell proliferation via ARG1-mediated mechanisms (Kusmartsev and Gabrilovich, 2005). In mice that are subcutaneously injected with CT26 colon cancer cells, single treatment with a small molecule ARG1 inhibitor (CB-1158) or an anti-PD-L1 antibody suppresses the tumor growth, and their tumor suppressive effect is enhanced by combining these two inhibitors (Steggerda et al., 2017). Similarly, in mice that have received orthotopic injection of 4T1 mammary tumor cells, the anti-PD1/anti-CTLA4 treatment in combination with CB-1158 significantly reduces growth of the primary tumor and decreases the number of lung metastases (Steggerda et al., 2017). These results suggest that CSF1R signaling may yield immune suppressive phenotype to TAM by inducing ARG1 expression in addition to support TAM accumulation in the tumor, and that addition of antagonists for CSF1R and/or ARG1 to checkpoint therapies can be a promising strategy.

A recent study demonstrates that expression of *Arg1* and *Tgfb* mRNA in TAM is significantly reduced by genetic depletion or pharmacological inhibition of phosphoinositide 3-kinase γ (PI3Kγ) in the mammary tumors developed in Polyoma Middle T oncogene (PyMT) transgenic mice, as well as the subcutaneous tumors established by LLC lung cancer cells in syngeneic C57BL/6 mice (Kaneda et al., 2016). These results indicate that PI3Kγ is another important regulator of immune suppressive phenotype of TAM. Interestingly, T cells isolated from the LLC tumors in PI3Kγ deficient mice are more cytotoxic than those in wild type mice. Furthermore, treatment with a PI3Kγ inhibitor (TG100-15) significantly augments the tumor suppressive effects of anti-PD1 antibody in a mouse model of head and neck squamous carcinoma (Kaneda et al., 2016). These data suggest that PI3Kγ inhibitors promotes cytotoxic capacity of T cell responses by blocking immune suppressive functions of TAM, and thus is useful to enhance therapeutic effects of checkpoint antagonists.

The immune suppressive features of macrophages within the tumor can also be interfered by inhibition of class IIa histone deacetylase (HDAC), enzymes that regulate activity of many transcription factors (Di Giorgio et al., 2015). In the mammary tumors developed in PyMT transgenic mice, a selective class IIa HDAC inhibitor (TMP195) switches dominant macrophage populations in the tumor from TAM to highly phagocytic macrophages, which suppresses tumor growth (Guerriero et al., 2017). Importantly, the treatment with TMP195 in combination with anti-PD1 antibody further reduces tumor burden in this model, whereas a single treatment with anti-PD1 antibody is not sufficient to suppress tumor development. Therefore, the class IIa HDAC inhibitor has a potential to enhance checkpoint therapy by drawing anti-tumor functions from tumor-infiltrating macrophages (Guerriero et al., 2017).

It is reported that the macrophage polarization to an immunosuppressive phenotype is also regulated by cytokines such as IL-4, IL-10, and IL-13 (Sica and Bronte, 2007). In cultured human macrophages, IL-10 induces key macrophage receptors (Ley et al., 2016) including toll-like receptors, Fc receptors (e.g., FcγR), and macrophage receptor with collagenous domain (MARCO) (Park-Min et al., 2005). Although their contribution to the immunosuppressive phenotype of TAM is not known yet, recent studies suggest some of these receptors as targets for the improvement of checkpoint therapies. For example, in mice with melanoma established by subcutaneous injection of B16 cells, an anti-MARCO monoclonal antibody treatment enhances the efficacy of anti-CTLA4 antibody treatment in suppressing tumor growth (Georgoudaki et al., 2016). In the B16 tumors as well as human melanoma samples, MARCO is predominantly expressed by TAM. Furthermore, anti-MARCO antibody treatment reduces the percentage of a distinct TAM population (known as M2 macrophage) that is reported to express ARG1 and suppress *in vitro* T cell proliferation (Movahedi et al., 2010; Georgoudaki et al., 2016). These results suggest that anti-MARCO antibody can switch TAM phenotype from the immunosuppressive to immune activating one, and thereby promotes anti-tumor activities of cytotoxic T cells. However, precise mechanisms behind the synergistic effects of anti-MARCO on anti-CTLA4 antibody treatment need to be further elucidated.

Another potential target is Fc-gamma receptor (FcγR), a receptor of immunoglobulin. In mice that are subcutaneously injected with MC38 colon cancer, a single treatment with anti-PD1 antibody can suppress tumor growth whereas the response to this therapy typically varies among animals. In contrast, addition of FcγR blocking antibodies to the anti-PD1 treatment completely suppresses tumor growth in all mice (Arlauckas et al., 2017). Interestingly, the intravital microscopy of the tumor has identified that anti-PD1 antibodies that initially bind to T cells are transferred to TAM in the tumor by 24 h, and the treatment with FcγR blocking antibodies prolongs the binding of anti-PD1 antibodies to tumor-infiltrating T cells (Arlauckas et al., 2017). These data indicate that TAM in the tumor microenvironment limit the efficacy of checkpoint therapy by depriving the antibodies against checkpoint receptors/ligands, and that inhibition of the interaction between Fc region of checkpoint blocking antibodies and Fc receptors in TAM can be a therapeutic option to improve the therapy.

Accumulating evidences indicate that TAM is the one of the major immune suppressor cell types in the solid tumors and that pharmacological interventions of TAM accumulation and/or function are promising strategies to improve checkpoint therapies. Although therapeutic effects of the TAM intervention/checkpoint inhibition combination therapy is evident from the previous pre-clinical studies, further basic researches will be required to apply this novel strategy to the clinic.

## Conclusion and future perspectives

**Figure 1:**
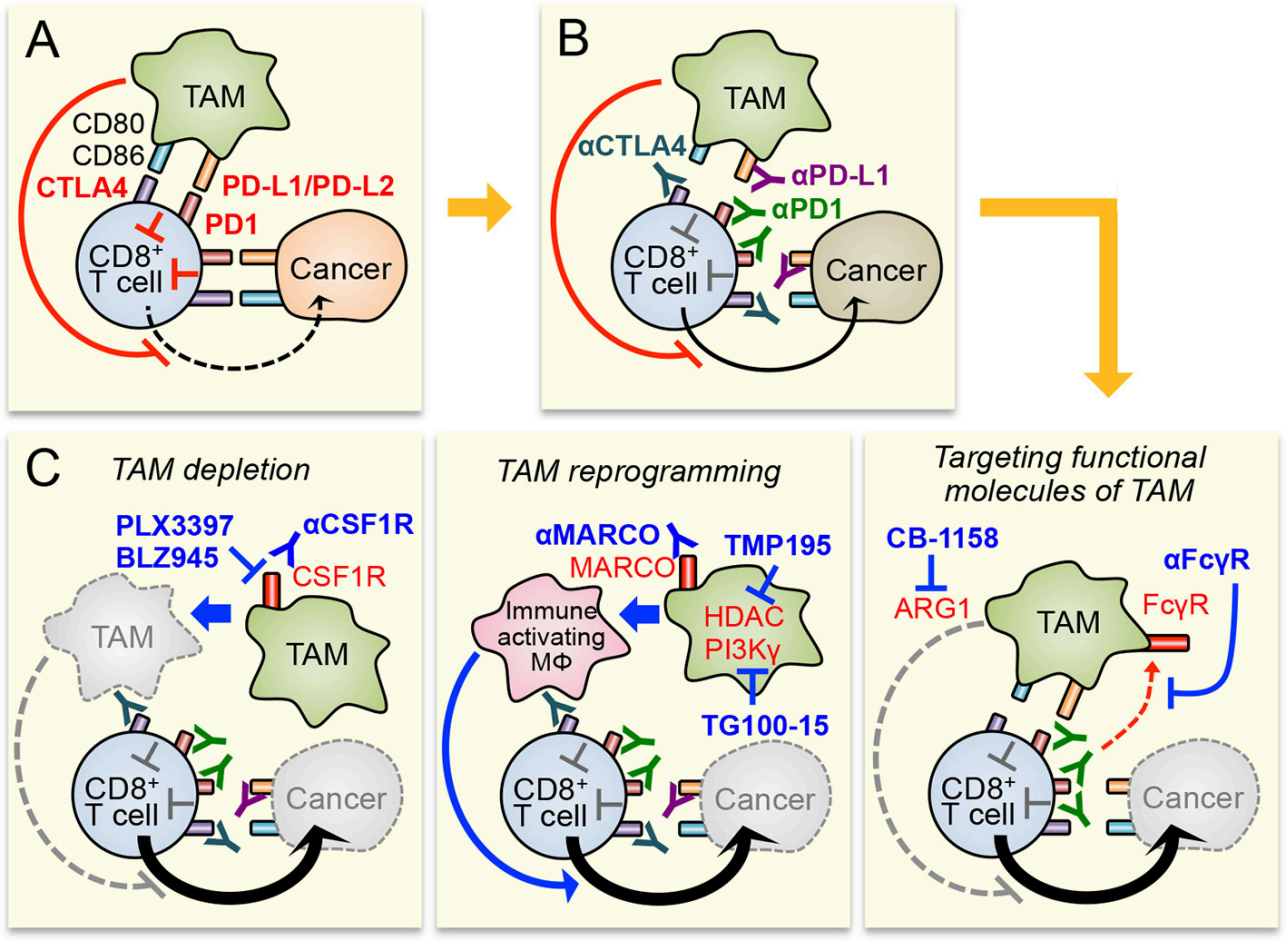
Potential therapeutic strategies to enhance immune checkpoint inhibitors by targeting tumor-associated macrophages (TAM). **(A)** Cytotoxicity of CD8^+^ T cell in the tumors is suppressed by immune checkpoint pathways activated by cancer cells and TAM. TAM also suppresses CD8^+^ T cell functions via checkpoint pathway independent mechanisms that are still under investigation. **(B)** Blockade of immune checkpoint pathway by antibodies for CTLA4, PD1, and PD-L1 enhances CD8^+^ T cell cytotoxicity. However, immune suppressive tumor microenvironment, especially TAM in it, will limit the anti-tumor efficacy of the checkpoint inhibitors. **(C)** Therapeutic efficacy of checkpoint inhibitors can be improved by TAM targeting through different strategies, i.e., TAM depletion (left), TAM reprogramming (central), and targeting functional molecules of TAM (right). MΦ means macrophage.

Accumulating evidences indicate that TAM is one of the major components of the immune suppressive tumor microenvironment, and is an attractive target to improve responses to immunotherapies. Therefore, several TAM targeting strategies (e.g., TAM depletion, TAM reprogramming, and targeting functional molecules of TAM) have been proposed to enhance efficacy of the immune checkpoint inhibition, one of the most promising immunotherapy for the treatment of solid tumors (Figure 1). Preclinical studies have suggested that the combination of these strategies with checkpoint inhibitors can enhance the therapeutic responses at least in melanoma and tumors in the lung, colon, and breast. Despite the encouraging preliminary results, all these strategies need further investigation before being applied for clinic as a combination therapy with checkpoint inhibitors.

The total depletion of monocytes and macrophages by CSF1R inhibitors is a straightforward and efficient approach (Mantovani et al., 2017). However, this strategy is not TAM specific and may cause high toxicity if patients are treated for prolonged periods (Cannarile et al., 2017). Moreover recent studies suggest that normal monocytic cells are required for T cell-mediated immune reaction, and thus the full depletion of monocytes and macrophages may not be ideal to combine with checkpoint inhibitors. Namely, classical monocytes (precursors of recruited macrophages including TAM) seem to be required for better responses to anti-PD1 therapy (Krieg et al., 2018) and macrophage-mediated T_reg_ cell depletion can co-define the efficacy of anti-CTLA4 therapy (Simpson et al., 2013). A potential approach alternative to the “total” depletion would be the “pulsing” ablation of TAM followed by recovery periods during which monocytes can return into the tumors and promote initial anti-tumor immune reactions before turning to TAM. However, this attractive strategy requires more knowledge about a timing of pulsing and immune interactions ongoing in all phases of tumor formation (Gil Del Alcazar et al., 2017). Another alternative approach to overcome potential issues in monocyte/macrophage depletion would be the targeting of cancer-associated immature myeloid cells (or monocytic-MDSC) that possess intrinsic immunosuppressive functions *in vitro* and give rise to TAM in tumors (de Haas et al., 2016; Kitamura et al., 2018; Veglia et al., 2018). Since gene expression profile of these TAM progenitor cells is distinct from that of normal monocytes (Kitamura et al., 2018; Veglia et al., 2018), targeting the progenitors would block TAM-mediated immunosuppression without affecting normal monocyte functions and thus improve checkpoint inhibitor more efficiently. The challenge of this approach is to identify specific markers for the progenitors, which will allow the selective targeting of a source of immunosuppressive myeloid cells.

The second promising strategy for TAM targeting is the reprogramming of TAM from immune suppressive and trophic cell to immune activating and tumoricidal one. However the extreme level of macrophage plasticity (Sica and Mantovani, 2012) will cause a potential risk of this strategy, i.e., the macrophages existing in the tumors can switch back to pro-tumor TAM when the reprogramming treatment is interrupted. The majority of studies published so far did not fully investigate the long-term effects of the TAM reprogramming agents after the treatment interruption, and thus more studies that fully elucidate the phenotype of macrophages after reprogramming are required for clinical application of this strategy.

The third TAM intervention strategy is to target functional molecules of TAM. An encouraging example is the blockade of Fc receptors on TAM that prevents deprivation of anti-PD1 antibodies and thereby enhances the efficacy of the checkpoint therapy (Arlauckas et al., 2017). However, Fc receptor inhibition may negatively affect another type of immunotherapy since myeloid cells and cytotoxic lymphocytes also express Fc receptors and require the receptors for antibody-mediated phagocytosis or antibody-dependent cellular cytotoxicity. Indeed, it has been reported that anti-CTLA4 antibody exerts the therapeutic effect through FcγR dependent T_reg_ cell depletion by macrophages and loss of FcγR reduces therapeutic response to CTLA4 therapy in mice with B16 melanoma (Simpson et al., 2013). It will be thus fundamental to identify TAM specific targets (such as MARCO) to improve therapy specificity of this TAM targeting strategy.

These potential issues for each TAM targeting strategy suggest that the next challenges in the tumor immunology field will be to identify specific markers and tailor the targeting only of the tumor promoting macrophage subpopulations in different cancers and cancer subtypes. The extensive use of single cell RNA sequencing, multiplex immunohistochemistry and mass cytometry techniques will considerably enhance our knowledge on the heterogeneity of TAM in tumors and define the selection of novel TAM targets for the improvement of cancer immunotherapy.

## Author contributions

LC and TK: conceptualized this review, decided on the content, and wrote the manuscript; TK: prepared the figure. All authors approved the final version of the manuscript and agreed to be accountable for all aspects of the work.

### Conflict of interest statement

The authors declare that the research was conducted in the absence of any commercial or financial relationships that could be construed as a potential conflict of interest.
